# Factors predicting incarceration history and incidence among Black and Latino men who have sex with men (MSM) residing in a major urban center

**DOI:** 10.1371/journal.pone.0265034

**Published:** 2022-03-08

**Authors:** Nina T. Harawa, Katrina M. Schrode, Joseph Daniels, Marjan Javanbakht, Anna Hotton, Solomon Makgoeng, Amy Ragsdale, John Schneider, Kayo Fujimoto, Robert Bolan, Pamina Gorbach

**Affiliations:** 1 Department of Medicine, David Geffen School of Medicine, University of California Los Angeles, Los Angeles, CA, United States of America; 2 Department of Epidemiology, Fielding School of Public Health, University of California Los Angeles, Los Angeles, CA, United States of America; 3 Department of Psychiatry and Human Behaviors, College of Medicine, Charles R. Drew University of Medicine and Science, Los Angeles, CA, United States of America; 4 Department of Medicine, University of Chicago, Chicago, IL, United States of America; 5 Chicago Center for HIV Elimination, Chicago, IL, United States of America; 6 Division of Health Promotion and Behavioral Sciences, University of Texas Health Science Center at Houston, Houston, TX, United States of America; 7 Department of Health Services, Los Angeles LGBT Center, Los Angeles, CA, United States of America; University of North Carolina at Chapel Hill, UNITED STATES

## Abstract

We analyzed data from a cohort of Black and Latino men who have sex with men (MSM) in order to identify correlates of prevalent and incident incarceration, including potential predictors related to their status as sexual and gender minorities (SGMs). Baseline and follow-up self-administered survey data were examined from Los Angeles County participants’ ages 18–45 years at enrollment who were either HIV negative or living with HIV, but recruited to over represent men who used drugs and men with unsuppressed HIV infection. Multivariable logistic regression models were developed to identify predictors of baseline incarceration history and of incident incarceration over study follow-up among 440 and 338 participants, respectively. Older age, Black race, low socioeconomic status, homelessness, stimulant use, and depression symptoms were associated with baseline incarceration history. The only SGM-related factor associated with baseline incarceration history was having experienced violence based on sexual orientation identity. Just one statistically significant, independent positive predictor of incident incarceration was identified: prior incarceration, whereas having four or more friends that could lend money was a statistically significant protective factor against incident incarceration. Fundamental Cause Theory provides a useful framework to explain identified predictors of incarceration. Addressing poverty, housing instability, inadequate access to health care, and their root causes is critical to reducing incarceration rates in this population, as is expanded access to both diversion and anti-recidivism programs and to evidence-based treatment for stimulant use disorders.

## Introduction

Black men of all sexual orientations and gay- and bisexually identified men of all race/ethnicities experience higher rates of incarceration compared with the general U.S. population [1, 2]. Approximately 5% of gay and bisexual men experience incarceration in any given year in the U.S. This rate is 1.57 times that of other men [2, 3]. These increased risks may be attributed to increased police surveillance of sexual and gender minority (SGM) individuals and venues, harsher punishments of SGM based on societal stigma and norms, and differential patterns of substance use [2, 4, 5]. Annual incarceration rates for Black gay, bisexual, and other men who have sex with men (hereafter, MSM) are even higher than are those observed for MSM as a whole (e.g., estimated at 31% in a multi-city study of those at increased risk for HIV infection or poor engagement in HIV care) [3, 6]. The umbrella label sexual and gender minority or SGM includes gender diverse populations and cis-gender individuals with same-gender attractions, some of whom are also attracted to other genders. The MSM describes cisgender men who have sex with men (MSM) and may identify with a range of orientations and communities. We use it when referring specifically to these men. When referring to factors and issues applying to SGM subpopulations more broadly, particularly those due to their status as minoritized communities, we use the term SGM.

Factors contributing to increased risk of incarceration in Black and Latino MSM as a group are multifold. Racism and xenophobia compound the homophobia many MSM of color experience. Racist and anti-immigrant rhetoric is common in many settings and media platforms [7, 8]. Immigration policy and practice has led to disproportionate incarceration and deportation of immigrants who are Black, Latino, or Muslim [8]. For MSM, deportation may return them to countries where it is illegal to be LGBTQ identified [9]. Such criminalization threat is compounded by restricted access to employment and to certain government resources, like Medicaid, limiting the ability of undocumented immigrants to build the social capital and socioeconomic standing that reduce incarceration risk [8, 10, 11]. Low access to quality education and living wage employment in many Black and Latino communities reduce the potential of financial independence and housing stability and contribute to behaviors that increase risk for both incarceration and negative health outcomes [12]. Compounding this incarceration risk, racially biased practices and policies in policing and criminal justice have resulted in higher rates of surveillance, searches, arrests, charges, or sentences for Black and Latino people compared with White people [13–16].

Once in the criminal justice system, SGM experience some of the highest rates of victimization, in addition to other experiences that may have negative long-term health implications [17–20]. Studies also show MSM report higher rates of solitary confinement and harsher punishments compared with cis-gender heterosexual people in custody [2]. In a study of people living with HIV who had been released from jail, it was shown that young Black MSM living with HIV were less likely than other men to receive interventions designed to improve HIV disease management while incarcerated [21]. Victimization and medical neglect during incarceration can exacerbate trauma, depression, and anxiety, thereby reducing future health care utilization [17–19, 22]. Victimization, co-occurring with social isolation and experiences of racism/xenophobia in the mainstream gay community has been shown to increase negative coping behaviors such as substance use following incarceration in Black and Latino MSM [21, 23, 24]. In short, these experiences can inhibit MSM’s health-protective behaviors post-release, leading to increased risk of morbidity and recidivism following incarceration [1, 21].

Criminal justice involvement (CJI) itself is inextricably linked to engagement in services–substance use treatment, harm reduction, HIV prevention and care, mental health, and gender affirmation—for conditions that disproportionately affect SGM populations [25–27]. However, despite their over representation among people with CJI and the increased levels of HIV, STIs, hepatitis, and substance use disorders in CJI populations, few policies and interventions to reduce recidivism or to address these conditions have been tailored for MSM with CJI [28]. Furthermore, research focused on predictors of incarceration among MSM is limited, with most of this literature limited to Black MSM. The paucity of information on which MSM of color are most affected by our system of corrections offers little guidance on what types of interventions or policy changes have the greatest potential for meeting the health needs of this group and for reducing their risk of incarceration [2].

These intersections of incarceration risk may be best understood by applying *Fundamental Cause Theory*. This theory holds that health disparities are determined by the degree to which someone has access to basic and innovative resources for health, well-being, and thriving [29–31]. Denied access to these resources results from systemic de-investment in communities in areas like education, information technology infrastructure, and business and organizational development for employment opportunity (e.g., redlining), and from punitive immigration and zero tolerance policies. Together these result in blocked pathways to employment, income, and wealth [32, 33]. This de-investment can set-up cumulative deficits across the life-course that contribute to health and other risks, including CJI, leading to negative outcomes [30]. Because this theory also articulates ways that disparities are recreated, even following the implementation of public health interventions, its objective is both to identify causes of disparities and to provide implications for potential approaches to preventing negative outcomes [34].

Given the over representation of Black and Latino MSM in the criminal justice system nationally [1, 2, 6], the large jail population in Los Angeles County [35], and the confluence of health and incarceration risk factors in Los Angeles (e.g., high levels of poverty and homelessness [36, 37], over 150,000 children referred to child welfare each year [38], and 22% of Black and 16% of Latino youth not finishing high school within five years [39]), we set out to examine predictors of incarceration history and incarceration incidence among Black and Latino participants in the MStudy—an ongoing prospective cohort study measuring factors linked to substance use and HIV transmission dynamics for MSM in Los Angeles, which focused on enrolling Black/African American and Latino/Hispanic MSM between 18 and 45 years [40]. Examination of factors associated with incarceration history is useful for identifying those factors that heighten risk of becoming incarcerated, as well as those that may stem from experiences of incarceration. Examination of factors associated with incident incarceration is useful for identifying factors that might heighten an individual’s risk of becoming incarcerated or reincarcerated in the future. We then apply a Fundamental Cause Theory Framework to discuss the implications of these predictors for life course CJI risk, and outline implications for interventions to reduce Black and Latino MSM’s incarceration risk.

## Methods

### Participants

Data were obtained from the ongoing Men who have Sex with Men & Substance Use Cohort at UCLA Linking Infections, Noting Effects (mSTUDY) that began enrolling participants in February 2015. MStudy enrolls people between the ages of 18 and 45 years at enrollment who were labeled male at birth and, if HIV-negative, reported condomless anal sex with a male in the prior 6 months. MStudy participants use computer-assisted self-interviews (CASI) to complete a baseline survey and then follow-up surveys every 6 months. HIV status at baseline is determined via the OraQuick® Advance Rapid HIV test with Western Blot confirmation.

Study recruitment approaches focused on cisgender men. They were designed to ensure a large sample of men that actively used substances other than alcohol or cigarettes, regardless of their HIV status, and to ensure that half of the total sample was living with HIV. Participants with HIV were recruited from a community-based clinic that offers comprehensive services for SGMs. HIV-negative participants were recruited through a community-based university research clinic using a range of approaches, including social media and distribution of study materials at sites for substance abuse treatment and other community settings. Additional study details can be found here [40]. All participants provided written informed consent after meeting with a study interviewer, reviewing the study objectives and procedures, and having any questions answered. The UCLA Institutional Review Board approved the mSTUDY protocols.

Racial/ethnic identity was determined from a question asking participants to “select the SINGLE race with which you most closely identify,” after they completed standard Office of Management and Budget race and ethnicity questions. Participants were also able to write in their own responses. At the time of analysis, there were 544 participants enrolled in mSTUDY. Although the study recruitment was focused on Black and Latino MSM, 104 MSM of other race/ethnicities were enrolled, but excluded from this analysis. Participants who wrote in a response indicating a mixed Black and Latino identity rather than selecting a single race were also excluded, because our models compared these groups. The current analysis focuses on the 440 Black/African American and Latino/Hispanic participants enrolled between February 2015 and November 2018 and followed for up to 4 years.

### Outcomes

The outcome variables, *history of incarceration at baseline* and *incident incarceration*, were based on responses to the question, “How many times have you been incarcerated for more than 24 hours?” which was asked at both the baseline and follow up surveys. History of incarceration was defined as having been ever incarcerated at baseline, while incident incarceration was defined as any incarceration during follow-up. Any response that was greater than zero on the baseline survey indicated a history of incarceration. Whereas, a higher number of incarcerations reported in a follow-up survey compared with baseline indicated incident incarceration. Participants with no follow-up visits were excluded from the analysis of incident incarceration.

Responses to the question on respondents’ total number of incarcerations were often inconsistent across follow-up interviews. They fluctuated up and down over time, rather than just increasing for those who recidivated or staying the same for those who did not (for example, reporting 4 total incarcerations at baseline and only 2 total incarcerations at the next visit). Therefore, we were not able to apply a repeated measures analysis to examine whether changes in potential predictors over follow-up led to increased incarceration risk. Hence, we employed additional, conservative criteria to define incident incarceration. Participants who reported a higher number of incarcerations at one follow-up interview but not subsequent interviews were excluded from the analysis. An exception was made if the only increase was reported at the most recent follow-up, as the respondent had no opportunity to include that incarceration at subsequent interviews. In June 2018, the incarceration question was revised for the follow-up surveys to ask only about the number of incarcerations *since the previous study interview*. For the few participants asked this question, we considered any response greater than zero to indicate incident incarceration. The sample for incident incarceration is smaller than that for history of incarceration due to the exclusions described above.

### Predictors

An initial, base set of independent covariates from the baseline survey was selected based on literature indicating that they have been shown to be predictors of incarceration [24, 34, 36, 41–43]. These included sociodemographic variables, social support, sexual behavior, substance use, and psychosocial measures. *Sociodemographic variables* included age, race/ethnicity, socioeconomic status (SES), and health insurance coverage. SES was defined as a composite variable with three levels based on self-reported annual income and homelessness in the prior month: 1) individual income less than $20,000 who experienced homelessness, 2) individual income less than $20,000 who did not experience homelessness, and 3) individual income of at least $20,000 (none of whom experienced homelessness). We created this composite variable to avoid collinearity between income and homelessness. The responses to these two variables formed three distinct clusters, which we used to define the categories of the composite variable. An income of $20,000 was the closest income category to the median pre-incarceration annual income (in 2014 dollars) reported by Black ($17,625) and Latino ($19,740) adult state prisoners in a study by the Prison Policy Institute [44].

*Psychosocial/behavioral measures* included social support, sexual and substance use behaviors, experiences of racism, and depression symptoms. The survey had a small set of questions related to *social support*. Based on our examination of bivariate associations with incarceration, we included a covariate for one question, “How many people can you borrow $50 from today?” We included two covariates relating to *sexual behavior*—total number of partners and reporting concurrent sexual partners in the last 6 months. These behaviors have been positively associated with activities that are criminalized, such as commercial sex and substance use, or that may signal a need to engage in survival sex, which is often a response to poverty or substance misuse. For *substance use*, we included frequency of binge drinking and types of other substances used in the prior 6 months, categorizing the latter as none, marijuana only, other non-stimulant drugs only (e.g., opioids, hallucinogens, ecstasy, synthetic drugs), or any stimulant drugs (i.e., methamphetamine, speed, powder or crack cocaine). Stimulant use was examined separately from other drugs because of several studies indicating that it has associations with incarceration and other negative outcomes that are stronger than and different than those observed with other types of illegal substances [45, 46].

Having experienced racism was measured with a five-point Likert scale in response to the questions, “In the past 12 months, how many times have you thought you have been treated rudely or unfairly because of your race or ethnicity?” and “In the past 12 months, how many times have you felt uncomfortable in a crowd of white gay men because of your race or ethnicity?” Item scores were summed to create a total score that was examined in bivariate analysis as a dose-response but dichotomized for analysis as any vs. none because strong outliers appeared to drive the association with incarceration. Depressive symptoms were assessed using the Center for Epidemiologic Studies *Depression (CES-D) scale, with scores of 20 and higher indicating symptom levels consistent with depression. This cutoff has been shown to have improved specificity among both HIV-negative individuals and those living with HIV [47].* Prior incarceration was included as an independent variable in analyses of incident incarceration.

To investigate potential predictors of incarceration specific to participants’ status as SGMs, we examined 8 additional variables. These variables encompass experiences and attributes that are far more common among SGMs than among cisgender heterosexual populations, including some that are not limited to MSM. This set comprised HIV status, sexual identity (based on reported self-reported sexual orientation and gender of sexual partners), internalized homophobia, specific sexual behaviors, and experiences of violence discrimination related to sexual orientation. *Internalized homophobia* was based on a 9-item measure, involving a 5-point Likert scale; because there is no defined clinical cutoff for the score, we categorized the summed scores based on tertiles of the possible sums [48]. *Sexual behaviors* included were number of male anal sex partners (last 6 months) and any exchange sex (last 3 months). Three variables captured experiences of verbal, physical, or sexual *violence* attributed to the participant’s sexual identity. We included total number of partners and concurrent sexual partners under *psychosocial/ behavioral measures*, as they are not specific to SGM populations. Having multiple or concurrent partners is moderately common in both heterosexual and SGM populations, whereas as selling sex is rare among heterosexual cisgender men and much more common among both cisgender MSM and transgender women. Of course, a cisgender man having anal sex with another man is specific to sexual minority men.

### Analysis

We first used chi-squared tests to examine bivariate relationships between the dependent variables of lifetime and incident incarceration and each of the independent variables, and then constructed a multivariable model. For each outcome, we included variables from the base-set of potential predictors with a p-value of ≤0.20 in bivariate analysis in a multivariable logistic regression model. Next, we dropped any base-set variables that yielded multivariable regression p-values > 0.20 before adding to the model all the SGM status-related variables that fit the same criterion (bivariate p≤ 0.20). Because mSTUDY enrollment was ongoing, the amount of time that analyzed participants had been in the study ranged from 4.5 months to 4 years. To account for this, we included the length of time between each respondents’ baseline and most-recent follow-up survey as a covariate in all models of incident incarceration. Bivariate statistics are based on all available data, resulting in slightly different sample sizes for some variables due to missing data. We used listwise deletion for regression models, which resulted in somewhat smaller sample sizes for these analyses than for bivariate analyses. We observed little change in the final models when they were refit using robust variance, indicating there were no issues of heteroscedasticity. To test goodness-of-fits of the models, we used Hosmer-Lemeshow tests that test for significant deviations between the modeled and observed outcomes; a p-value < 0.05 indicates significant deviation and therefore a poor fit. We report adjusted odds ratios (ORs) and 95% confidence intervals (CIs) from the final models for incarceration history and incident incarceration. We carried out all analyses in SAS 9.2.

We conducted two sensitivity analyses. To determine the impact of our conservative criterion for incident incarceration, we re-ran the final incidence model after including the participants who has been excluded because they reported a higher number of incarcerations at only one follow-up visit (n = 30). To explore whether predictors of incarceration differed by gender expression, in the second sensitivity analysis, we re-ran the final models for both lifetime and incident incarceration after excluding participants who described themselves with terms other than male (n = 52). These participants included individuals who identified with other terms (e.g., realness, butch queen, crossdresser) or who refused any gender label.

## Results

### Lifetime incarceration

Results of the bivariate chi-squared tests are presented in Table 1. Those with incarceration histories were predominately over the age of 24 (89%); 61% were Black, and 54% were living with HIV. Those of the lowest SES were the most likely to have an incarceration history at 51%, while only 11% of those of the highest SES reported incarceration histories (p<0.001 for overall differences). Eighty-three percent of those with a history of incarceration had public health insurance, compared with 59% of those that had not been incarcerated. However, fewer participants with a history of incarceration were uninsured (8% vs. 17%; p<0.001). More of those with a history of incarceration were stimulant users than were those without an incarceration history (73% vs. 44%, p<0.001). Just 17% of those with a history reported not using any substances in the last 6 months. Depressive symptoms were more common in those who had experienced incarceration, as was exchange sex, and reported experiences of physical violence related to sexual identity. Chi-squared tests indicated there were no statistically significant differences in history of incarceration or any of the predictors between participants with and without follow-up visits (results not shown).

**Table 1:** Association between examined potential predictors and incarceration among Black/ African American and Hispanic/Latino mSTUDY participants.

* *	History of Incarceration (n = 440*)			Incident Incarceration (n = 338*)		
	No (n = 278)	Yes (n = 162)		No (n = 279)	Yes (n = 59)	
	% (n)	% (n)	*p-value*	% (n)	% (n)	*p-value*
***Potential base-set predictors***						
Sociodemographic						
**Age at baseline**			0.001			0.135
18–24	23 (63)	11 (18)		22 (60)	12 (7)	
25–34	49 (136)	46 (75)		47 (131)	46 (27)	
35–45	28 (79)	43 (69)		32 (88)	42 (25)	
**Race/Ethnicity**			0.013			0.133
Black/African American	49 (136)	61 (99)		52 (145)	63 (37)	
Hispanic/Latino/Spanish	51 (142)	39 (63)		48 (134)	37 (22)	
**Socio-economic status (SES)**			< .001			0.059
Lowest SES	12 (30)	21 (31)		13 (33)	24 (13)	
Middle SES	66 (164)	75 (112)		71 (178)	69 (38)	
Highest SES	22 (54)	5 (7)		16 (40)	7 (4)	
**Health insurance**			< .001			0.034
Private health insurance	24 (65)	9 (15)		21 (59)	10 (6)	
Public health insurance	59 (163)	83 (132)		64 (177)	81 (48)	
No health insurance	17 (47)	8 (13)		15 (41)	8 (5)	
**Previous incarceration**						< .001
No	-	-		68 (191)	42 (25)	
Yes	-	-		32 (88)	58 (34)	
Psychosocial factors						
**Social support (how many people do you think you can borrow $50 from today?)**			0.534			0.009
0	28 (74)	33 (53)		25 (70)	46 (26)	
1	13 (34)	13 (21)		13 (37)	14 (8)	
2–3	22 (58)	23 (37)		23 (63)	19 (11)	
4+	38 (102)	31 (51)		39 (109)	21 (12)	
**Total number of partners in last 6 mos.**			0.935			0.452
0 or 1	20 (55)	19 (31)		20 (54)	21 (12)	
2–5	27 (75)	26 (42)		28 (77)	35 (20)	
6+	53 (144)	54 (87)		53 (145)	44 (25)	
**Sex with concurrent partners in last 6 mos.**			0.373			0.650
No	51 (133)	47 (67)		50 (129)	54 (28)	
Yes	49 (127)	53 (77)		50 (127)	46 (24)	
**Binge drinking in last 6 mos. (6 or more drinks/occasion)**			0.064			0.030
Never	37 (101)	46 (75)		39 (108)	51 (29)	
Monthly or less	50 (137)	38 (62)		49 (136)	30 (17)	
Daily to weekly	14 (38)	15 (25)		13 (35)	19 (11)	
**Drug use last 6 mos.**			< .001			0.005
No drug use	36 (99)	17 (28)		35 (97)	16 (9)	
Marijuana only	13 (35)	7 (11)		11 (30)	5 (3)	
Any stimulant use	44 (122)	73 (119)		48 (134)	74 (42)	
Use of non-stimulant drugs only	7 (20)	2 (4)		6 (18)	5 (3)	
**Experienced racism in past 12 mos.**			0.533			0.419
Never	40 (110)	43 (69)		43 (120)	37 (22)	
At least once	60 (168)	57 (93)		57 (159)	63 (37)	
**Depressive symptom score**			< .001			0.050
Symptoms not consistent with depression (<20)	65 (180)	48 (77)		61 (171)	47 (28)	
Symptoms consistent with depression (≥ 20)	35 (98)	52 (85)		39 (108)	53 (31)	
***Potential predictors associated with status as a sexual and gender minority (SGM)***						
**Sexual orientation**			0.083			0.587
Attraction to or sex with men only	86 (239)	80 (129)		84 (235)	81 (48)	
Attraction to or sex with men and other genders	14 (39)	20 (33)		16 (44)	19 (11)	
**HIV status**			0.058			0.249
Positive	45 (125)	54 (88)		48 (133)	56 (33)	
Negative	55 (153)	46 (74)		52 (146)	44 (26)	
**Internalized homonegativity**			0.563			0.043
First tertile	73 (203)	69 (111)		75 (210)	59 (35)	
Second tertile	22 (60)	25 (41)		20 (56)	34 (20)	
Third tertile	5 (14)	6 (10)		5 (13)	7 (4)	
**How many men have you had anal sex with in the last 6 mos.?**			0.281			0.361
0	10 (28)	8 (13)		9 (25)	10 (6)	
1–5	47 (132)	57 (92)		54 (151)	53 (31)	
6–10	20 (55)	18 (29)		18 (49)	25 (15)	
11+	23 (63)	17 (28)		19 (54)	12 (7)	
**Engaged in exchange sex in last 3 mos.**			0.012			0.067
No	83 (224)	73 (115)		82 (225)	71 (40)	
Yes	17 (46)	27 (43)		18 (49)	29 (16)	
**Experienced verbal violence related to sexual identity in past 12 mos.**			0.188			0.669
No	57 (156)	51 (81)		54 (148)	57 (33)	
Yes	43 (117)	49 (79)		46 (127)	43 (25)	
**Experienced physical violence related to sexual identity in past 12 mos.**			< .001			0.147
No	75 (208)	56 (89)		71 (195)	61 (36)	
Yes	25 (68)	44 (71)		29 (81)	39 (23)	
**Experienced sexual violence related to sexual identity in past 12 mos.**			0.933			0.433
No	91 (247)	91 (144)		93 (254)	90 (52)	
Yes	9 (25)	9 (15)		7 (20)	10 (6)	

*Sample size may differ slightly for some variables due to missing data, the level of which ranges 0–9%.

Variables associated with an increased odds of having an incarceration history in the multivariable logistic model (n = 378; Table 2) were age group 35–46 (AOR = 2.57 95% CI: 1.17–5.64), Black race (AOR = 1.99 95% CI: 1.21–3.28), lowest and middle vs. higher SES (AOR = 3.91; 3.95 95% CI: 1.39–10.97; 1.63–9.60 respectively), stimulant use vs. no substance use (AOR = 3.17 95% CI: 1.74–5.79), and having depressive symptoms (AOR = 1.72 95% CI: 1.04–2.87). Odds of incarceration were similar in those who only used marijuana compared to those who did not use any substances. Exchange sex and HIV status were not statistically significantly associated with incarceration history in the adjusted model. Having experienced physical violence related to sexual identity (AOR = 2.09 95% CI: 1.19–3.69) was the only SGM-related factor independently associated with incarceration history in the logistic model.

**Table 2:** Potential predictors* of history of incarceration at baseline among Black/African American and Hispanic/Latino mSTUDY participants (n = 378^#^).

	AOR [95% CI]	p-value
***Base-set model predictors***		
**Age at baseline**		
18–24	Ref	Ref
25–34	1.78 [0.85–3.72]	0.127
35–46	2.57 [1.17–5.64]	0.019
**Race/Ethnicity**		
Hispanic/Latino/Spanish	Ref	Ref
Black/African American	1.99 [1.21–3.28]	0.007
**Socio-economic status**		
Lowest SES	3.91 [1.39–10.97]	0.010
Middle SES	3.95 [1.63–9.60]	0.002
Highest SES	Ref	Ref
**Health insurance**		
No insurance	0.65 [0.23–1.84]	0.422
Public health insurance	1.77 [0.85–3.68]	0.124
Private health insurance	Ref	Ref
**Drug use last 6 mos.**		
No drug use	Ref	Ref
Marijuana only	1.47 [0.58–3.75]	0.420
Any stimulant use	3.17 [1.74–5.79]	<0.001
Only non-stimulant drugs	0.78 [0.21–2.90]	0.714
**Depression symptom score**		
Not consistent with depression (<20)	Ref	Ref
Consistent with depression (≥20)	1.72 [1.04–2.87]	0.036
***Predictors associated with status as an SGM***		
**Sexual orientation**		
Attraction to or sex with men only	Ref	Ref
Attraction to or sex with men and other genders	1.29 [0.66–2.54]	0.453
**HIV status**		
Negative	Ref	Ref
Positive	0.87 [0.51–1.49]	0.621
**Engaged in exchange sex in last 3 mos.**		
No	Ref	Ref
Yes	1.10 [0.61–1.98]	0.754
**Experienced verbal violence related to sexual identity in past 12 mos.**		
No	Ref	Ref
Yes	0.77 [0.44–1.34]	0.353
**Experienced physical violence related to sexual identity in past 12 mos.**		
No	Ref	Ref
Yes	2.09 [1.19–3.69]	0.011

*Model controls for all covariates shown in table.

^#^Sample size may differ slightly for some variables due to missing data.

SGM = sexual and gender minority.

AOR = adjusted odds ratio.

CI = confidence interval.

A Hosmer-Lemeshow (H-L) goodness of fit test gave no evidence of poor model fit (p = 0.421).

Sensitivity analysis excluding individuals who only used non-male terms to describe themselves yielded similar findings, with the exception that depressive symptoms no longer had a statistically significant association with incarceration history (AOR = 1.54 95% CI: 0.90–2.64). Other results are not shown.

### Incident incarceration

Age, race/ethnicity, SES, and health insurance coverage showed similar bivariate associations with incident incarceration as with lifetime incarceration, although only health insurance status reached statistical significance (Table 1). Fifty-eight percent of those with incident incarceration had a history of incarceration compared with just 32% of those with no incident incarcerations (p<0.001). As with lifetime incarceration, most of those who experienced incident incarceration were stimulant users (74%; p = 0.005). Those with incident incarceration were more likely to report never binge drinking than those without incident incarceration (51% vs. 39%, p = 0.030). Those with incident incarceration appeared to lack social support; 46% reported that there was no one they could borrow $50 from vs. 25% of those without incident incarceration (p = 0.009). Of the SGM status-related variables, only internalized homonegativity reached statistical significance, with 41% of those with incident incarceration having scores in the second or third tertile compared with 25% of those without incident incarceration (p = 0.043).

After adjustment, predictors of incident incarceration were quite different from those found for incarceration history (n = 328; Table 3). In the first stage of variable selection, sociodemographic factors and substance type failed to meet the p-value ≤0.2 criterion. One SGM-related factor (internalized homonegativity) met this criterion. The strongest and only statistically significant positive predictor in the fully adjusted model was an incarceration history at baseline (AOR = 3.25; 95% CI: 1.68–6.28). However, having at least 4 friends that could lend them money was a statistically significant negative predictor (AOR = 0.43; 95% CI = 0.18–0.99), and infrequent binge drinking (compared with no binge drinking) neared statistical significance as a negative predictor (AOR = 0.53; 95% CI = 0.26–1.08).

**Table 3:** Potential predictors* of incident incarceration during follow-up among Black/African American and Hispanic/Latino mSTUDY participants (n = 328).

	AOR [95% CI]	p-value
***Base-set model predictors***		
**History of incarceration at baseline**		
No	Ref	Ref
Yes	3.25 [1.68–6.28]	<0.001
**Social support (how many people do you think you can borrow $50 from today?)**		
0	Ref	Ref
1	0.78 [0.30–2.07]	0.623
2–3	0.55 [0.24–1.29]	0.170
4+	0.43 [0.18–0.99]	0.049
**Binge drinking in last 6 months (6 or more drinks)**		
Never	Ref	Ref
Daily to weekly	1.12 [0.44–2.86]	0.808
Monthly or less	0.53 [0.26–1.08]	0.081
***Predictors associated with status as an SGM***		
**Homonegativity**		
First tertile	Ref	Ref
Second tertile	1.67 [0.81–3.42]	0.163
Third tertile	1.56 [0.36–6.79]	0.553
**Engaged in exchange sex in last 3 months**		
No	Ref	Ref
Yes	1.17 [0.55–2.49]	0.691
**Experienced physical violence related to sexual identity in past 12 mos.**		
No	Ref	Ref
Yes	0.94 [0.47–1.88]	0.854

*Model controls for all covariates shown in table and length of time followed in study.

SGM = sexual and gender minority.

AOR = adjusted odds ratio.

CI = confidence interval.

A Hosmer-Lemeshow (H-L) goodness of fit test gave no evidence of poor model fit (p = 0.274).

In both sensitivity analyses (results not shown), the confidence interval for the association between social support and incident incarceration widened and the effect was no longer significant (male only: AOR = 0.43; 95% CI = 0.17–1.06; inconsistent numbers of incarcerations: AOR = 0.51; 95% CI = 0.25–1.07). Although the association with binge drinking was unaffected by including individuals who reported inconsistent numbers of incarcerations, when the model was limited to people who solely described themselves as male, the effect estimate was stronger and statistically significant (AOR = 0.40; 95% CI = 0.18–0.85). SGM status-related factors remained statistically non-significant.

## Discussion

Consistent with prior research on the general U.S. population, we found that incarceration history was positively associated with public health insurance status, Black race/ethnicity, low income (with and without recent homelessness), recent stimulant use, and depression symptoms for Black and Latino MSM in Los Angeles [24, 26, 36, 41, 43]. Over 90% of the sample was low-income or had public or no health insurance, over 55% used stimulants, and 42% had symptoms consistent with depression. Contrary to expectations and the prior research with Black MSM that we outlined in the Introduction, HIV status, recent exchange sex, and self-reported experiences of racism did not have independent associations with history of incarceration [6]. Perhaps more surprising, no sociodemographic or psychosocial factor predicted incident incarceration in the adjusted models; however, financial social support was protective and previous incarceration associated with greatly increased risk. The fact that this study population comprised just Black and Latino MSM and was largely of low SES, provides important context to these findings and may explain why certain variables were not found to be protective.

Although gay and bisexually identified populations have been shown to have increased risks of incarceration compared with the general population [2], none of the SGM-related variables were associated with incident incarceration, and just one—physical violence related to sexual identity—was associated with incarceration history. A striking 32% of the population reported such violence in the prior 12 months. Although exposure to violence has been associated with increased risk of subsequent incarceration in other research [24, 38], it is also well documented that MSM experience high rates of sexual and physical violence within custody [18, 20]. Hence, some of the violent experiences reported may have occurred during, rather than prior to, the incarcerations MStudy participants reported at baseline. The potential physical and psychological trauma associated with such violence should be a consideration for clinicians serving this population. For example, it may lead some MSM to avoid seeking care for HIV or other STIs during periods of incarceration for fear of disclosure of their sexuality and resulting increased risk of violence.

The high prevalence of the incarceration predictors that we identified in this sample points to potentially synergistic socioeconomic forces that disproportionately impact Black and Latino MSM and is consistent with fundamental cause theory [29, 30]. Specifically, one’s socioeconomic standing, as defined as wealth, social connections, knowledge and power, influences access to resources and to technical developments that determine the degree to which individuals can mitigate or navigate emerging risk factors over time [31, 34]. Without such access, disparities in both disease and incarceration rates persist, even as individuals receive interventions (e.g., public health insurance or HIV transitional case management) to mitigate some of these risks [29]. These findings highlight the strong association between access to resources and incarceration history and indicate that prior incarceration heightens risk for subsequent incarceration—pointing to a cycle that individuals often struggle to leave once they enter. For example, bail amounts increase with prior convictions; bidirectional associations between CJI and lower SES are well documented; and policies that prevent access to social services or education to those with convictions contribute to recidivism [43, 49].

Stimulant use stands out from other types of substances in its well-documented associations with HIV risk [40, 50] and here in its strong association with incarceration history [36, 50]. Nevertheless, adjusting for incarceration history in our analysis attenuated its association with incident incarceration. Given that the mean age of study participants at baseline was 31 years (SD = 6.8), we surmise that most participants who both used stimulants and were vulnerable to incarceration would have experienced it prior to baseline hence its lack of a significant association with incident incarceration. The negative association of both infrequent binge drinking and social support with incident incarceration that we found may stem from infrequent drinkers having employment and disposable income. For example, some men gather to drink on paydays where they socialize, share knowledge, and make connections. In their comprehensive review of the literature, Peele and Brodsky identify these and other potential benefits associated with moderate drinking [51]. The fact that our measure of social support was based on the ability of a friend to lend money provides a further association with socioeconomic standing. Social support can provide access to resources for increasing socioeconomic standing (e.g., through job leads) and for circumventing CJI risk. For example, someone with friends who are willing and able to lend them money is more likely than someone without such friends to have a person that they can call on to help bail them out of jail, pay a legal fine, or drive to pick them up if they are in a situation that may lead to legal trouble.

### Limitations

Our analysis is based on a non-random sample of MSM participants in a long-term study who were recruited to meet specific enrollment targets. Although the population included 52 who are not male identified, the study was not intended to represent those who did not identify as male. The different criteria and approaches for recruiting participants living with HIV and HIV-negative participants may have obscured associations with HIV status. Finally, the self-reported data had inconsistencies in new incarcerations over time, prohibiting analysis of potential time-varying predictors and raising concerns about validity. To confirm that study participants had experienced incident incarceration, we compared data from a sample of MSTUDY participants (n = 22) to the L.A. County Sheriff Department’s database on bookings into custody (http://app5.lasd.org/iic/). All who met our criteria for incarceration during follow-up had at least one recorded incarceration during the period, increasing confidence that we were able to capture accurately which participants experienced incident incarceration. Our study’s focus on Black and Latino MSM limits our ability to examine the impact of self-reported experiences of racism or homophobia as White and heterosexual men are not available for comparison. We anticipate lower endorsements of racism and internalized negativity about sexual orientation in White MSM or similar race, heterosexually identified men who have sex with women, respectively. Such a sample would be more likely to reveal associations of racism and homophobia with incarceration.

Finally, Los Angeles legalized medical marijuana use in 1996 and recreational use in 2016; hence, our findings may not generalize to areas that have not decriminalized marijuana use or time periods preceding decriminalization [52, 53]. Consistent with research in this and a similar Los Angeles study population, there were no positive associations between marijuana use and incarceration, risky behaviors, or negative health outcomes [40]. Findings are inconsistent with a similar analysis that our group conducted in a Chicago cohort of young Black transgender women and MSM, where we found increased odds of incident incarceration in participants who used marijuana only compared with participants who did not use substances [41]. Prior to January 2020, even medical marijuana was illegal in Illinois. The illegality of marijuana use, which has limited impact on community and personal safety, can generate unnecessary pathways into CJI [24, 41, 54].

### Public health implications

Fundamental cause theory highlights the pernicious ways that risks realign to recreate disparities when downstream rather than upstream, fundamental causes of negative outcomes are addressed. Viewed from this lens, structural- and individual- interventions, like providing alternatives to incarceration for drug offenses, making harm-reduction services broadly available, bail reform, and decriminalization of the low-level offenses that often entrap people who are homeless in the criminal justice system, cannot be implemented in isolation. They must be paired with more systematic initiatives to address fundamental causes through expanded access to living wage employment and investments into both low-income housing and educational/curricular renewal (K-12, higher education, professional training) that also integrate technical innovations in order to successfully address disparities in incarceration risk. These efforts should be shaped by an understanding of how institutionalized racism has contributed to current disparities in both SES and CJI [12, 15]. The associations of incarceration with sexual orientation-related victimization in this study and other research showing high levels of exposure to community violence among Black MSM with CJI [1], further highlight the need for custody- and community-based services that are trauma-informed and competent in serving the needs of SGM reentry populations.

### Conclusion

Given the strong associations of incarceration history with poverty, homelessness, Black race/ethnicity, and violence related to sexual identity in this sample of Black and Latino MSM, addressing root causes is critical to any effort to reduce risk of both incarceration and its negative sequelae. Root causes like anti-Black racism, xenophobia, gender- and hetero- normativity, and income inequality create social conditions that place individuals at increased risk of both poor health and incarceration. The strong associations of incarceration history with stimulant use and depression symptoms point to a need for increased access to evidence-based treatment for substance use and mental health disorders both in custody and in communities. Efforts to divert people from incarceration to these services are also key but may not be successful without other fundamental changes. While many of the predictors of incarceration for MSM and other SGM may not differ from those of cis-gender heterosexual people, their risk of incarceration is higher, as is their risk of sexual assault and abuse while in custody. This heightens the urgent need for tailored structural-, and individual-level interventions to reduce these risks and to address the unique needs of SGMs with incarceration histories.

## References

[1] Schneider JA, Lancki N, Schumm P. At the intersection of criminal justice involvement and sexual orientation: Dynamic networks and health among a population-based sample of young Black men who have sex with men. Soc Networks. 2017;51:73–87. Epub 2017/10/25. doi: 10.1016/j.socnet.2017.04.001 29062165 PMC5650246

[2] Meyer IH, Flores AR, Stemple L, Romero AP, Wilson BD, Herman JL. Incarceration rates and traits of sexual minorities in the United States: National Inmate Survey, 2011–2012. Am J Public Health. 2017;107(2):267–73. Epub 2016/12/21. doi: 10.2105/AJPH.2016.303576 27997242 PMC5227944

[3] Herman J RS, Mottet L, Anafi M. The Report of the 2015 U.S. Transgender Survey. Washington, DC: National Center for Transgender Equality. 2016.

[4] Luhur W, Meyer I, Wilson B. Policing LGBQ People. Los Angeles, CA: The Williams Institute, 2021.

[5] Stewart-Winter T. Queer Law and Order: Sex, criminality and policing in the late twentieth-century United States. J Am Hist. 2016;102:61–72.

[6] Brewer RA, Magnus M, Kuo I, Wang L, Liu TY, Mayer KH. Exploring the relationship between incarceration and HIV among black men who have sex with men in the United States. J Acquir Immune Defic Syndr. 2014;65(2):218–25. Epub 2013/10/05. doi: 10.1097/01.qai.0000434953.65620.3d 24091691 PMC3898433

[7] Wilson RA, Land MK. Hate speech on social media: Content moderation in context. Conn L Rev. 2020;52:1029.

[8] Taylor TN, DeHovitz J, Hirshfield S. Intersectional stigma and multi-level barriers to HIV testing among foreign-born Black men from the caribbean. Front Public Health. 2019;7:373. Epub 2020/01/31. doi: 10.3389/fpubh.2019.00373 31998675 PMC6965168

[9] African Commission on Human and Peoples’ Rights, Inter-American Commission on Human Rights, United Nations, University of Pretoria. Faculty of Law. Ending violence and other human rights violations based on sexual orientation and gender identity: A joint dialogue of the African Commission on Human and People’s Rights, Inter-American Commission on Human Rights and United Nations Pretoria, South Africa: Pretoria University Law Press (PULP); 2016. i, 85 pages p.

[10] Valera P, Boyas JF. Perceived social ties and mental health among formerly incarcerated men in New York City. Int J Offender Ther Comp Criminol. 2019;63(10):1843–60. Epub 2019/03/05. doi: 10.1177/0306624X19832239 30829090

[11] Kipke MD, Kubicek K, Akinyemi IC, Hawkins W, Belzer M, Bhandari S, et al. The Healthy Young Men’s Cohort: Health, stress, and risk profile of Black and Latino young men who have sex with men (YMSM). J Urban Health. 2020;97(5):653–67. Epub 2020/08/31. doi: 10.1007/s11524-019-00398-6 32864727 PMC7560671

[12] Bailey ZD, Feldman JM, Bassett MT. How structural racism works—racist policies as a root cause of U.S. racial health inequities. N Engl J Med. 2021;384(8):768–73. Epub 2020/12/17. doi: 10.1056/NEJMms2025396 33326717 PMC11393777

[13] Bowleg L, Maria Del Rio-Gonzalez A, Mbaba M, Boone CA, Holt SL. Negative police encounters and police avoidance as pathways to depressive symptoms among US Black men, 2015–2016. Am J Public Health. 2020;110(S1):S160–S6. Epub 2020/01/23. doi: 10.2105/AJPH.2019.305460 31967888 PMC6987946

[14] Knox D LW, Mummulo J. Administrative records mask racially biased policing. Am Polit Sci. 2020;114:619–37.

[15] Bishop ET HB, Obiofuma C, Owusu F. Racial Disparities in the Massachusetts Criminal System. Boston: Criminal Justice Policy Program Havard Law School, 2020.

[16] Kutateladze BL AN, Johnson BD, Spohn CC. Cumulative disadvantage: Examining racial and ethnic disparity in prosecution and sentencing. Criminology. 2014;52(3):514–51. 10.1111/1745-9125.12047.

[17] Bacak V, Thurman K, Eyer K, Qureshi R, Bird JDP, Rivera LM, et al. Incarceration as a health determinant for sexual orientation and gender minority persons. Am J Public Health. 2018;108(8):994–9. Epub 2018/06/22. doi: 10.2105/AJPH.2018.304500 29927654 PMC6050838

[18] Jenness V MC, Matsuda K, Sumner J. Violence in California Correctional Facilities: An empirical examination of sexual assualt. Center for Evidence-Based Corrections: UC Irvine, May 16, 2007. Available from: https://cpb-us-2.wpmucdn.com/sites.uci.edu/dist/0/1149/files/2013/06/PREA_Presentation_PREA_Report_UCI_Jenness_et_al.pdf.

[19] Boxer P MK, Delorenzo T. Exposure to violent crime during incarceration: Effects of psychological adjustment following post-release. Crim Justice Behav. 2009;36(8):793–807. 10.1177/0093854809336453.

[20] Beck A HP, Berzofsky M, Caspar R, Krebs C. Sexual Victimization in Prisons and Jails Reported by Inmates, 2011-12-Update. The Bureau of Justice Statistics, 2014 December. Report No.: NCJ Number 241399. Available from: https://bjs.ojp.gov/library/publications/sexual-victimization-prisons-and-jails-reported-inmates-2011-12-update.

[21] Vagenas P, Zelenev A, Altice FL, Di Paola A, Jordan AO, Teixeira PA, et al. HIV-infected men who have sex with men, before and after release from jail: The impact of age and race, results from a multi-site study. AIDS Care. 2016;28(1):22–31. Epub 2015/08/15. doi: 10.1080/09540121.2015.1062464 26275122 PMC4713253

[22] 2020 Greater Los Angeles Homeless Count shows 12.7% rise in homelessness despite sustained increase in number of people rehoused. Los Angeles Homeless Services Authority. Published June 12, 2020 | Last updated September 03, 2020. Report No. Available from: https://www.lahsa.org/news?article=726-2020-greater-los-angeles-homeless-count-results.

[23] Turpin R, Khan M, Scheidell J, Feelemyer J, Hucks-Ortiz C, Abrams J, et al. Estimating the roles of racism and homophobia in HIV testing among Black sexual minority men and transgender women with a history of incarceration in the HPTN 061 Cohort. AIDS Educ Prev. 2021;33(2):143–57. Epub 2021/04/07. doi: 10.1521/aeap.2021.33.2.143 33821677 PMC10576191

[24] Hotton A, Quinn K, Schneider J, Voisin D. Exposure to community violence and substance use among Black men who have sex with men: examining the role of psychological distress and criminal justice involvement. AIDS Care. 2019;31(3):370–8. Epub 2018/10/04. doi: 10.1080/09540121.2018.1529294 30280579 PMC6382567

[25] Harawa NT, Amani B, Rohde Bowers J, Sayles JN, Cunningham W. Understanding interactions of formerly incarcerated HIV-positive men and transgender women with substance use treatment, medical, and criminal justice systems. Int J Drug Policy. 2017;48:63–71. Epub 2017/08/15. doi: 10.1016/j.drugpo.2017.05.013 28804052 PMC5620016

[26] Zaller N, Brinkley-Rubinstein L. Incarceration, drug use, and infectious diseases: a syndemic still not addressed. Lancet Infect Dis. 2018;18(12):1301–2. Epub 2018/11/06. doi: 10.1016/S1473-3099(18)30538-3 30385159

[27] Brewer R, Ramani SL, Khanna A, Fujimoto K, Schneider JA, Hotton A, et al. A systematic review up to 2018 of HIV and associated factors among criminal justice-involved (CJI) Black sexual and gender minority populations in the United States (US). J Racial Ethn. 2021:1–46. Epub 2021/07/24. doi: 10.1007/s40615-021-01076-7 34296420 PMC8297427

[28] Harawa NT, Brewer R, Buckman V, Ramani S, Khanna A, Fujimoto K, et al. HIV, sexually transmitted infection, and substance use continuum of care interventions among criminal justice-involved Black men who have sex with men: A systematic review. Am J Public Health. 2018;108(S4):e1–e9. Epub 2018/11/02. doi: 10.2105/AJPH.2018.304698 30383433 PMC6215366

[29] Chang VW, Lauderdale DS. Fundamental cause theory, technological innovation, and health disparities: The case of cholesterol in the era of statins. J Health Soc Behav. 2009;50(3):245–60. Epub 2009/08/29. doi: 10.1177/002214650905000301 19711804 PMC2885132

[30] Link BG, Phelan J. Social conditions as fundamental causes of disease. J Health Soc Behav. 1995;Spec No:80–94. Epub 1995/01/01. 7560851

[31] Emerson AM, Carroll HF, Ramaswamy M. Education level as a predictor of condom use in jail-incarcerated women, with fundamental cause analysis. Public Health Nurs. 2018;35(4):273–80. Epub 2018/05/29. doi: 10.1111/phn.12514 29806134 PMC6053914

[32] Alson JG, Robinson WR, Pittman L, Doll KM. Incorporating measures of structural racism into population studies of reproductive health in the United States: A narrative review. Health Equity. 2021;5(1):49–58. Epub 2021/03/09. doi: 10.1089/heq.2020.0081 33681689 PMC7929921

[33] Li KC. The private insurance industry’s tactics against suspected homosexuals: redlining based on occupation, residence and marital status. Am J Law Med. 1996;22(4):477–502. Epub 1996/01/01. 9006667

[34] Phelan JC, Link BG, Tehranifar P. Social conditions as fundamental causes of health inequalities: theory, evidence, and policy implications. J Health Soc Behav. 2010;51 Suppl:S28–40. Epub 2010/12/22. doi: 10.1177/0022146510383498 20943581

[35] Miaschi J. The Largest Jails in the United States. WorldAtlas, 2017 September 28. Available from: https://www.worldatlas.com/articles/the-largest-jails-in-the-united-states.html.

[36] Anderson-Carpenter KD, Fletcher JB, Swendeman D, Reback CJ. Associations between sociodemographic characteristics and substance use disorder severity among methamphetamine-using men who have sex with men. Subst Use Misuse. 2019;54(11):1763–73. Epub 2019/05/12. doi: 10.1080/10826084.2019.1610445 31075997 PMC6644069

[37] Anderson-Carpenter KD, Fletcher JB, Reback CJ. Associations between methamphetamine use, housing status, and incarceration rates among men who have sex with men and transgender women. J Drug Issues. 2017;47(3):383–95. Epub 2017/07/04. doi: 10.1177/0022042617696917 28670005 PMC5485860

[38] Severe M, Scheidell JD, Dyer TV, Brewer RA, Negri A, Turpin RE, et al. Lifetime burden of incarceration and violence, internalized homophobia, and HIV/STI risk among Black men who have sex with men in the HPTN 061 Study. AIDS Behav. 2021;25(5):1507–17. Epub 2020/08/17. doi: 10.1007/s10461-020-02989-w 32797357 PMC8022355

[39] California Department of Education. 2018–19 Five-Year Cohort Graduation Rate: Los Angeles County Report Sacramento, CA. 2021. Available from: https://dq.cde.ca.gov/dataquest/.

[40] Gorbach PM, Javanbakht M, Shover CL, Bolan RK, Ragsdale A, Shoptaw S. Associations between cannabis use, sexual behavior, and sexually transmitted infections/human immunodeficiency virus in a cohort of young men who have sex with men. Sex Transm Dis. 2019;46(2):105–11. Epub 2019/01/15. doi: 10.1097/OLQ.0000000000000919 30640212 PMC6342487

[41] Hotton AL, Chen YT, Schumm P, Khanna AS, Brewer R, Skaathun B, et al. Socio-structural and neighborhood predictors of incident criminal justice involvement in a population-based cohort of young Black MSM and transgender women. J Urban Health. 2020;97(5):623–34. Epub 2020/03/18. doi: 10.1007/s11524-020-00428-8 32180129 PMC7560631

[42] Brewer RA, Magnus M, Kuo I, Wang L, Liu TY, Mayer KH. The high prevalence of incarceration history among Black men who have sex with men in the United States: associations and implications. Am J Public Health. 2014;104(3):448–54. Epub 2014/01/18. doi: 10.2105/AJPH.2013.301786 . 24432948 PMC3953792

[43] Wakefield S, Uggen C. Incarceration and stratification. Annu Rev Sociol. 2010;36:387–406. Epub April 20, 2010.

[44] Rabuy B, Kopf, D. Detaining the Poor. Northampton, MA: Prison Policy Initiative, 2016 May 10. Report No.: 10.

[45] Gjersing L, Bretteville-Jensen AL. Characteristics and risk of incarceration among "hard-to-reach" people who use drugs: A five-year prospective cohort study combining self-reports and registry data. Int J Drug Policy. 2021;95:103288. Epub 2021/05/19. doi: 10.1016/j.drugpo.2021.103288 34004380

[46] Weiser SD, Neilands TB, Comfort ML, Dilworth SE, Cohen J, Tulsky JP, et al. Gender-specific correlates of incarceration among marginally housed individuals in San Francisco. Am J Public Health. 2009;99(8):1459–63. Epub 2009/06/23. doi: 10.2105/AJPH.2008.141655 19542041 PMC2707486

[47] Armstrong NM, Surkan PJ, Treisman GJ, Sacktor NC, Irwin MR, Teplin LA, et al. Optimal metrics for identifying long term patterns of depression in older HIV-infected and HIV-uninfected men who have sex with men. Aging Ment Health. 2019;23(4):507–14. Epub 2018/02/10. doi: 10.1080/13607863.2017.1423037 29424569 PMC6085148

[48] Okafor CN, Gorbach PM, Ragsdale A, Quinn B, Shoptaw S. Correlates of preexposure prophylaxis (PrEP) use among men who have sex with men (MSM) in Los Angeles, California. J Urban Health. 2017;94(5):710–5. Epub 2017/06/11. doi: 10.1007/s11524-017-0172-z 28600749 PMC5610125

[49] Holzer HJ. Collateral costs the effects of incarceration on the employment and earnings of young workers. Bonn, Germany: IZA,; 2007. Available from: http://www.iza.org/en/webcontent/publications/papers/viewAbstract?dp_id=3118.

[50] Shoptaw S, Reback CJ. Methamphetamine use and infectious disease-related behaviors in men who have sex with men: implications for interventions. Addiction. 2007;102 Suppl 1:130–5. Epub 2007/05/12. doi: 10.1111/j.1360-0443.2006.01775.x 17493062

[51] Peele S, Brodsky A. Exploring psychological benefits associated with moderate alcohol use: a necessary corrective to assessments of drinking outcomes? Drug Alcohol Depend. 2000;60(3):221–47. Epub 2000/10/29. doi: 10.1016/s0376-8716(00)00112-5 11053757

[52] California Legislative Information. Control, Regulate and Tax Adult Use of Marijuana Act, 11362.1(c) (2016).

[53] California Legislative Information. Compassionate Use Act of 1996, Health and Safety Code 11362.5 (1996).

[54] Voisin DR, Hotton AL, Schneider JA, Team UCS. The relationship between life stressors and drug and sexual behaviors among a population-based sample of young Black men who have sex with men in Chicago. AIDS Care. 2017;29(5):545–51. Epub 2016/09/04. doi: 10.1080/09540121.2016.1224303 27590043 PMC5577924

